# Development of Scoring Functions for Antibody Sequence Assessment and Optimization

**DOI:** 10.1371/journal.pone.0076909

**Published:** 2013-10-21

**Authors:** Daniel Seeliger

**Affiliations:** Departement of Lead Identification and Optimization Support, Boehringer Ingelheim Pharma GmbH & Co. KG, Biberach/Riss, Germany; Universita’ di Padova, Italy

## Abstract

Antibody development is still associated with substantial risks and difficulties as single mutations can radically change molecule properties like thermodynamic stability, solubility or viscosity. Since antibody generation methodologies cannot select and optimize for molecule properties which are important for biotechnological applications, careful sequence analysis and optimization is necessary to develop antibodies that fulfil the ambitious requirements of future drugs. While efforts to grab the physical principles of undesired molecule properties from the very bottom are becoming increasingly powerful, the wealth of publically available antibody sequences provides an alternative way to develop early assessment strategies for antibodies using a statistical approach which is the objective of this paper. Here, publically available sequences were used to develop heuristic potentials for the framework regions of heavy and light chains of antibodies of human and murine origin. The potentials take into account position dependent probabilities of individual amino acids but also conditional probabilities which are inevitable for sequence assessment and optimization. It is shown that the potentials derived from human sequences clearly distinguish between human sequences and sequences from mice and, hence, can be used as a measure of *humaness* which compares a given sequence with the phenotypic pool of human sequences instead of comparing sequence identities to germline genes. Following this line, it is demonstrated that, using the developed potentials, humanization of an antibody can be described as a simple mathematical optimization problem and that the *in-silico* generated framework variants closely resemble native sequences in terms of predicted immunogenicity.

## Introduction

Owing to the extraordinary role antibodies play in life science research and in the pharmaceutical industry they are one of the most intensively studied class of proteins [1]. However, generation, manufacturing and storage of antibodies still poses challenges as many molecule properties like pharmacokinetics (PK), solubility, expression, viscosity and long-term stability are very difficult to predict or yet not predictable at all [2]–[5]. Although encouraging progress has been made in recent years to establish a rational link between sequence, structure and molecule properties our current understanding of these relationships is rather limited [6]–[11]. Statistical analyses of antibody sequences and the ability to distinguish between frequently occuring and rare sequence patterns therefore offer an alternative, knowledge-based approach to reduce developability risks by detecting unusual sequence patterns that have a potentially negative impact on the relevant properties. This becomes particularly evident if we regard the fact that the difference between a *well-behaved* antibody and a problematic one can be as small as one amino acid [12]–[15].

The majority of marketed antibodies and those in clinical trials are derived from natural B-cell repertoires of mice or mice with an engineered human germline repertoire [16]. In B-cells the genes encoding for the antibody are assembled from different gene fragments (termed V and J genes for the light chain, V,D and J genes for the heavy chain) and enzymes which randomly add and cut off nucleotides at the junctions account for additional diversity. In the subsequent affinity maturation cycles further mutations are randomly introduced in the varible domains of heavy and light chains which fine-tune the interactions with the antigen.

The entire process thus is a random, evolutionary process employing classical Darwinian mutation and selection. However, the selection criteria are defined by the organism that hosts the B-cell and it has to be noted that these selection criteria are of biological nature and not necessarily in line with biotechnological requirements. There is no evolutionary pressure on living organisms to select antibodies with a thermodynamic stability beyond 60 degrees, low aggregation tendency and low viscosity at concentration above 100 mg/ml. Accordingly animals do not optimize antibodies for properties that make them suitable to be put on the shelf for months.

An alternative source of antibodies are display technologies. Here, synthetic or semisynthetic libraries encoding either for the entire antibody, the antigen binding fragment (Fab) or only the variable domains (Fv) fused into a single chain (scFv) are cloned into surface proteins of yeast or phages [17]–[19]. This elegant fusion of proteins to their encoding genes enables an iterative cycle of in-vitro selection and optimization for binding. However, properties which are important for manufacturability are beyond the selection criteria just like for antibodies selected in-vivo and as a result antibodies, although optimally designed for their biological purpose, often fail to fulfil the demands of biotechnological manufacturing and the demands of being used as drugs in humans.

Although all of the biotechnologically relevant properties are encoded in the antibody sequence and gradual steps are taken to detect and rationally eliminate individual shortcomings, our capabilities to translate sequences into favourable CMC (chemical manufactoring control) and PK are at their infancy. Yet with the growing number of antibodies characterized on the protein level and the curation of sequence databases statistical methods can provide valuable insights into the phenotypes of *naturally matured* antibodies without comprehension of all the constraints leading to their selection. Of particular importance thereby are correlated mutations [20]. While two point mutations, if occuring individually, can be detrimental for protein stability, their concerted occurence may be neutral or even beneficial. These couplings in sequence alignments have been studied for very different purposes, often with a link to protein structure, function and evolution [21]–[26]. In variable domains (Fv) of antibodies such cooperative mutations are found as well [27], [28]. But although a functional driving force for some correlated positions can not be excluded for antibodies, most of the correlated mutations in antibodies appear through different underlying germline genes and, although the cooperativity is statistically significant, a structural or functional cause seems unlikely for most of them, which is underscored by the fact that correlations in human sequences differ from correlations in murine sequences. Consequently, it is not a subject of this paper to elucidate origins of cooperativity in antibody sequences but rather take correlations as a species specific fingerprint into account to develop heuristic scoring functions specific for antibodies from human and murine origin.

The variable domains (Fv) of antibodies show a substantial amount of variability, most pronounced in the complementary determining regions (CDRs) but also in the framework. The variability is partly encoded in the germline genes but many alterations arise from somatic hypermutation during affinity maturation. While amino acids located in the CDRs in most cases have some contribution to the binding affinity, the effect of mutations in the framework is highly diverse. Some do as well contribute to the binding affinity either by a direct interaction with the antigen or via a shaping and stabilizing affect on the conformation of the CDRs [29]. But many framework mutations are simply there as a consequence of random mutation with a neutral effect on potency but with a potentially detrimental effect on other molecule properties which affect late-stage pharmaceutical development.

The scoring functions developed in this paper specifically address this problem and provide a rational way to analyze sequences, identify potentially critical residues and to guide antibody engineering. In a second application it is shown how such potentials can be used in combination with optimization algorithms to sample *human-like* framework sequences which offers an alternative, stochastic approach for antibody humanization.

## Results and Discussion

### Development of Heuristic Scoring Functions

From a multiple sequence alignment (MSA) the probabilities 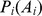 for finding amino acid *A* in position *i* can be readily obtained. Likewise the conditional probabilities 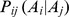 which describe the probability of finding amino acid *A* in position *i* when amino acid *A* is found in position *j* can be calculated.

If we describe a sequence as system of discrete states of its individual positions, a quantity 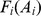 can be defined as a statistical energy for amino acid *A* at position *i* in the MSA. Using the inverse Boltzmann formula we can compute 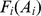 from the observed probability 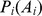 as
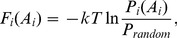
(1)where 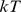 in this context denotes an artificial quantity which can be neglected. 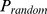 is the probability of finding amino acid *A* in a random setting and is set to 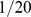 for simplicity. (Although the 20 amino acids are not equally distributed within the proteome using the proteome distributions to calculate 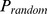 would not add accuray since the developed scoring functions are specifically designed for subtypes of antibody chains from different species which show different patterns of amino acid usage compared to the overall proteome.). For each conditional probability the statistical energy is accordingly



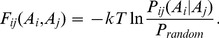
(2)Hence, the total score for a sequence of length *N*, omitting the prefactor 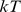, writes as
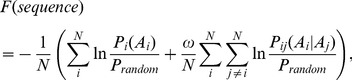
(3)where 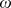 is a parameter which balances the contributions from individual and conditional probabilities. Since a major application of the scoring function is to guide sequence optimization of a given sequence an additional term is introduced which imposes a restraint towards a reference sequence, e.g. the starting sequence. To this end the sequence identity between the current sequence *S* and the reference sequence *R* is calculated and a potential of the form 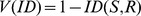, where ID is the sequence identity between zero and one, is used to restrain the sampling around the reference sequence. The complete scoring function thus writes
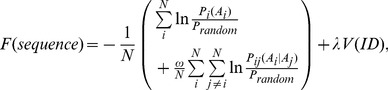
(4)where 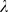 is a weighting factor to control the strength of the restraint. At many positions, the 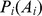 or 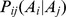 is zero. In these cases 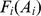 and 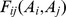 cannot be calculated using the logarithm which requires the use of pseudocounts. To this end the highest 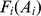 or 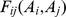, respectively, calculated from the lowest non-zero probability was taken in scaled with 1.1 to obtain an upper limit of contributions to the score from amino acids in a sequence which have zero-frequency at this position in the dataset of antibody sequences.

In total five different scoring functions have been developed for different antibody chain types from MSAs of publically available antibody sequences which were taken from the Absysis database [30], [31] (http://www.bioinf.org.uk/abysis/).

- F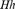: Heavy chain scoring function derived from human sequences.
- F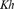: 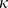-chain scoring function derived from human sequences.
- F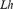: 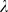-chain scoring function derived from human sequences.
- F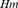: Heavy chain scoring function derived from murine sequences.
- F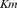: 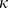-chain scoring function derived from murine sequences.

### Sequence Sampling and Optimization

The derived scoring functions can be used in combination with sampling algorithms to generate optimized sequences. For the present work a Monte Carlo protocol has been developed which employs two different *moves* to sample the sequence space. The first move is simply a random mutation at a random position, whereas the second attempts a double mutation at two random positions. The new sequence is evaluated with the scoring function and accepted with a probability 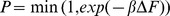, where 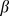, known as 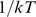 from the classical Metropolis criterion [32], is a parameter to adjust acceptance rate and sampling.

### Comparison of Human and Murine Sequences

**Figure 1:**
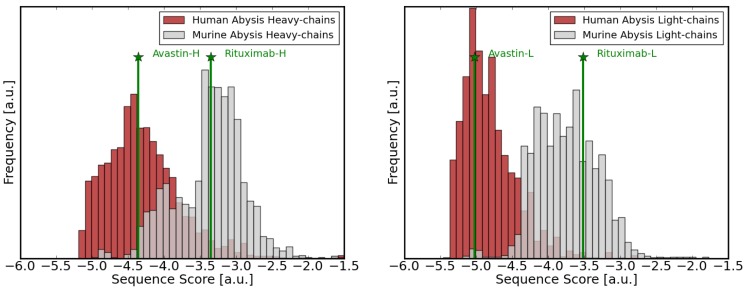
Comparison of human and murine sequences. Histograms of statistical sequence scores calculated for human (red) and murine (grey) sequences. Left: Heavy chains (F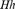). Right: Light chains (F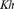). Scores for Rituximab, a chimeric antibody, and Avastin(Bevacizumab), a humanized antibody are indicated by green lines/stars.

The scoring function which has been derived from human antibody sequences can be used to evaluate sequences. Computation of the score essentially denotes a statistical comparison with all sequences that were used to develop the potential. Sequence patterns which only infrequently occur in human sequences thereby contribute larger values to the overall sequence score which has the consequence that sequences with several unusual patterns score high. This is for instance the case if we score sequences of murine origin with the potential that has been derived from human sequences. Figure 1 shows histograms of the F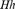 and F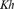 scores for human and murine heavy and 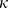-type sequences in the Abysis dataset [30], [31]. There is some overlap, as some murine germline genes are similar to human germlines, but the sequence score in most cases clearly distinguishes between sequences originating from either of the two species. Hence, the sequence score does not assess *humaness* in terms of sequence similarity with human germline genes but rather by a phenotypic comparison with a large dataset of human antibodies. This has the advantage that somatic mutations that frequently occur are less penalized by the score than accidential mutations never seen before. On the other hand, antibodies derived from rarely used germlines, even if 100% human, score higher. This is in fact closer to reality as the use of the different germlines in the antibodyome is far from equally balanced and certain germlines are heavily preferred over others. Moreover, in a recent large scale study it was impressively shown that certain germlines and heavy/light chain combinations consistently show higher expression levels and superiour biophysical properties, irrespective of the target they bind [33]. Therefore, in many cases there might be good reasons not to use the closest germline gene as a guide for sequence optimization.

**Figure 2:**
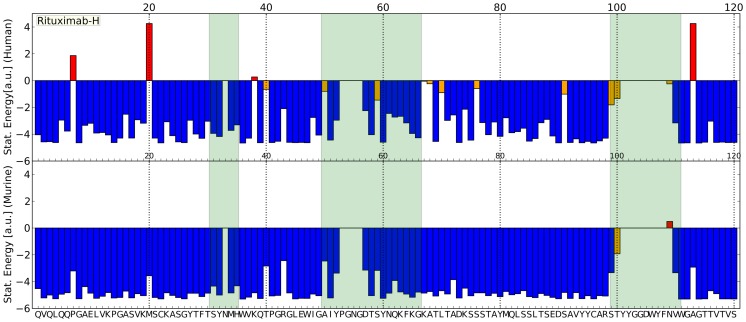
Sequence analysis of Rituximab-VH. Contributions to the total score are mapped onto individual residues. Yellow and red colors indicate that this amino acid is uncommon in this position and/or shows unfavourable couplings with other positions. Green shaded areas indicate CDRs of which some residues are not taken into account. Upper graph: Ritxumab heavy chain with human heavy chain potential (F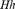). Lower graph: Rituximab heavy chain with murine heavy chain potential (F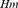).

If we use the derived score for sequence optimization the individual contribution of each amino acid to the overall score is more important than the total score as they reveal uncommon sequence patterns. Figure 2 shows the contributions of each residue in the Rituximab heavy chain to F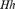 (upper graph) and F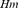 (lower graph). What becomes evident from the plot is that all amino acids contribute favourably to the murine score but that some positions are highly unusual in human sequences. This is the typical picture one gets for murine antibodies, however, if high-energy positions appear for a murine sequence in F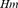 or for a human sequence in the F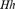, this serves as a warning flag and the respective positions should be carefully investigated and mutation to more favourably scoring amino acids should be considered.

### Stochastic Humanization

**Figure 3:**
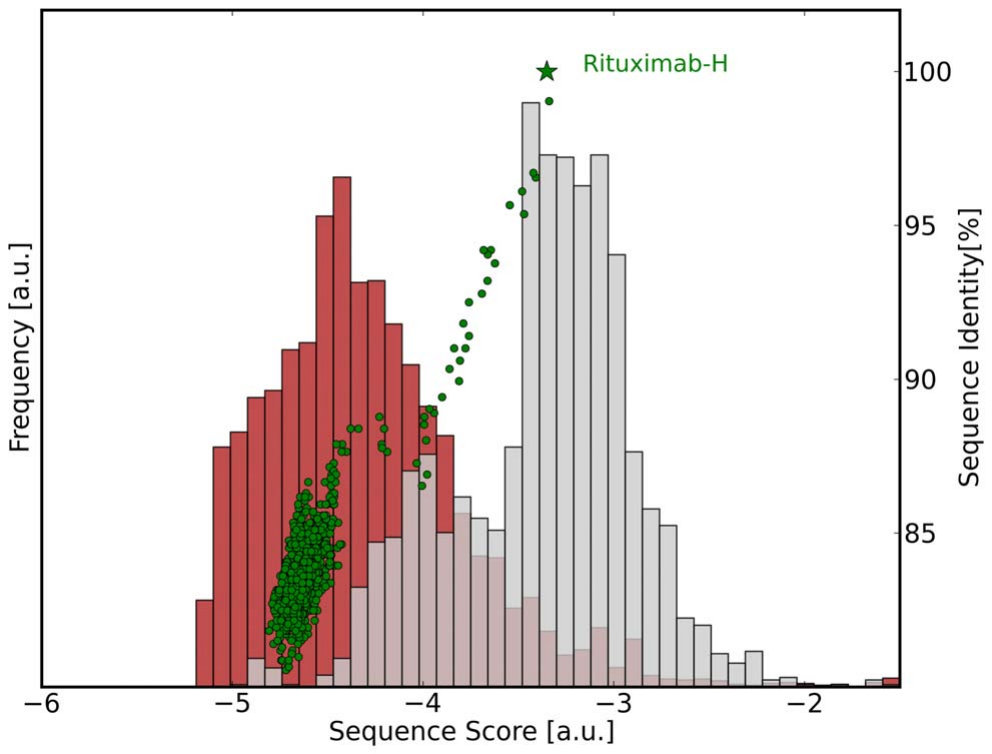
Stochastic Humanization of Rituximab-VH. Humanization of a murine heavy chain can be treated as an optimization of the objective function F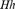. The Monte Carlo algorithm starts from the sequence of Rituximab-VH and optimizes the scoring function and the sequence identity to the parental sequence simultaneously.

As shown in the previous paragraph low sequence scores indicate sequences that consist of patterns commonly observed in human antibody sequences. From this observation it follows that the humanization of a murine sequence can be regarded as an optimization problem which can be addressed with stochastic methods like Monte Carlo Sampling. Humanization of an antibody sequence essentially means to find sequences which are as human as possible while staying as close as possible to the murine precursor [34]. This problem is described by equation 4 where the sequence identity to a reference sequence (in this case the parental murine sequence) is used to restrain the sampling. Figure 3 shows an application of the stochastic humanization to the heavy and light chains of Rituximab. The algorithm starts with the Rituximab sequence and optimizes the sequence score and sequence identity to the parent sequence simultaneously. Which property, the phenotypic humaness or the similarity to the parental sequence, dominates the sampling can be adjusted by the parameter 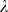 in equation 4. In the shown examples for this work 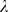 was set to 2 for all cases as this has been empirically found to be a reasonable choice. However, in practice the function of the antibody needs to be retained and there is no general procedure or parameter set to ensure optimal humaness and function, as there is in general no optimal way to humanize antibodies. Yet, the approach offers a rational way to alternatively humanized sequences beyond germlining and subsequent mutations back to the murine sequence.

Humaness is commonly expressed as percent sequence identity to the closest human germline or by statistical analysis of sequence identities to known human antibodies [35]. However, the actual purpose of humanization is not to increase sequence identity but to reduce immunigenicity. Although the immune response of an organism to a foreign protein is a complex process, T-cell mediated immunogenicity is to some extend predictable. If T-cell receptors recognize complexes between the major histocompatibility complex (MHC) II and peptides derived from the foreign proteins that are presented on the surface of antigen presenting cells (APC), the APC become activated which eventually leads to diversification and the secretion of antibodies against the foreign protein. If the foreign protein is a therapeutic antibody these antibodies are usually referred to as anti-drug antibodies (ADA). Appearance of such ADAs is generally unwanted since it may give rise to drug intolerance and compromise the therapeutic success. A first step in the T-cell mediated immune response is the loading of a peptide onto MHCII. The affinity of peptides derived from digested proteins to different MHCII allels is therefore a crucial prerequisite for an immune response and computationally predicted affinities of all possible T-cell epitopes derived from a protein serve as a surrogate parameter for the immunogenic potential of a protein. An algorithm which makes use of this approach and is frequently used to assess the immunogenicity risk of antibodies and other proteins is the Epivax software [36], [37]. The *tregitope adjusted Epivax Score*, calculated from a sequence, gives an estimate for the immunogenic potential of a protein.

**Figure 4:**
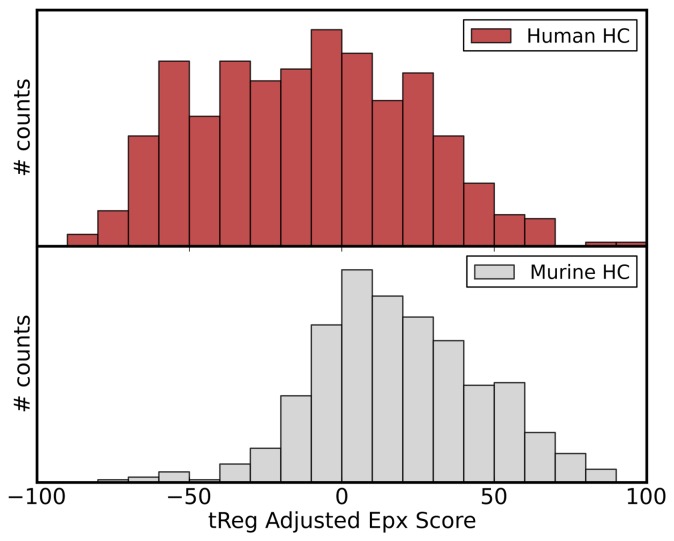
Predicted immunogenicity. Distribution of Epivax Scores for human and murine heavy chain sequences of 500 randomly picked sequences from the Abysis data set are shown. Low Scores indicate a lower risk of immunogenicity.

Figure 4 shows the *tregitope adjusted Epivax Score* computed for 500 randomly picked human and 500 randomly picked murine heavy chain sequences. Lower scores indicate low immunogenic potential and non-immunogenic antibodies usually have Epivax Scores below 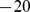, whereas antibodies known to induce immune responses in a substantial number of patients have Epivax scores greater than zero. What becomes evident when looking at the histograms is that the distribution of the scores is rather broad and that many human antibodies are predicted to be immunogenic which certainly is a tendency towards overprediction. Nevertheless, a humanized antibody should in any case display a lower Epivax score than its murine precursor. This at least is part of risk mitigation strategies in antibody development.

**Figure 5:**
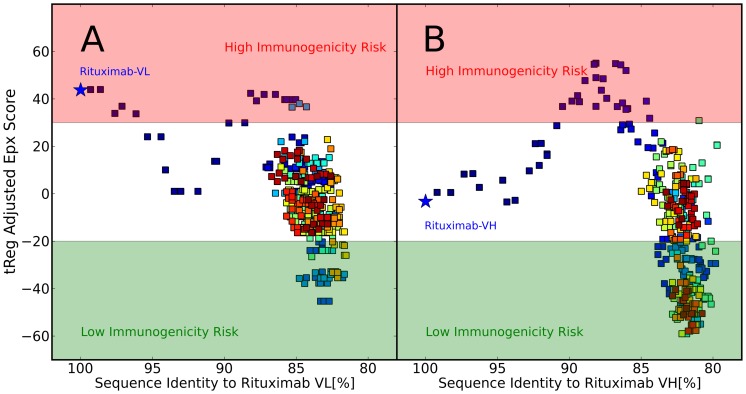
Development of Epivax Scores during stochastic humanization of Rituximab. A) Light Chain. B) Heavy Chain. Color codes indicate progress of the simulation, starting from blue to red. Both trajectories sample sequences with low immunogenic potential as predicted by the Epivax score.

To test the hypothesis, that sequences generated by the stochastic humanization procedure described above resemble *human-like* sequences, Epivax scores were calculated for the entire sequence trajectories. Figure 5 shows the development of the Epivax score over the course of Monte Carlo Simulations starting from the Rituximab light chain (A) and the Rituximab heavy chain (B) using F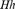 and F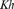, respectively, as functions to optimize. The values for the sequence scores are not shown in the plots but in figure 3 it can be seen that the generated sequences that show 80–85% sequence similarity to the parental chains appear in the bulk of the known human sequences and thus are considered human in the light of the scoring function. If we now look at the predicted immunogenicity of these sequences it becomes evident that the stochastic humanization protocol samples sequences with very low predicted immunogenic potential which strongly indicates that the generated sequences are in fact *human-like*. Hence, although immunogenicity as expressed by the Epivax score is not a quantity that is explicitely considered or optimized in the humanization protocol, it is implicitely encoded in the developed scoring functions and optimized as a side effect.

**Figure 6:**
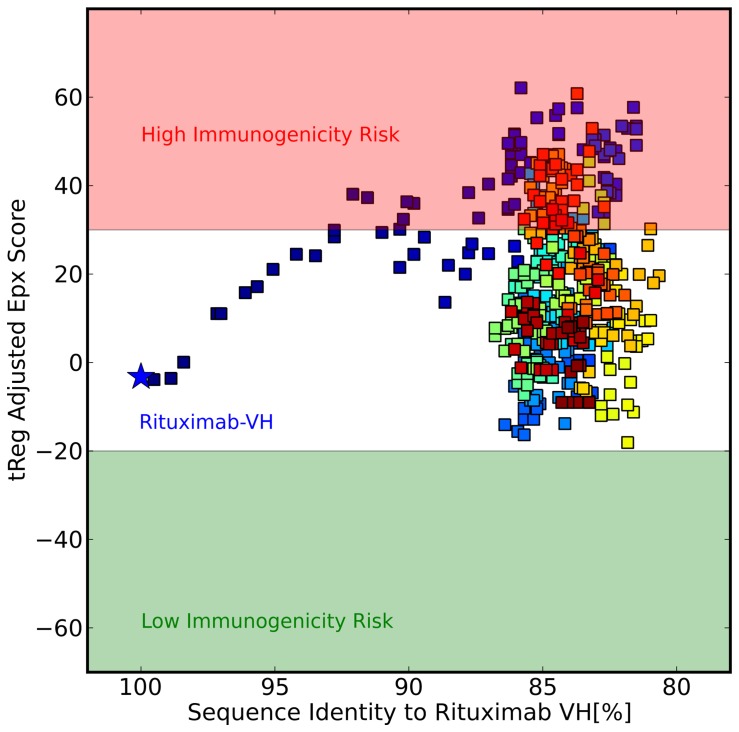
Couplings are required to generate non-immunogenic sequences. The plot shows the development of the Epivax score over the course of a Monte Carlo optimization of Rituximab-VH. The scoring function used here does not consider couplings of sequence positions and as a consequence, no sequences with low immunogenic potential are sampled.

**Figure 7:**
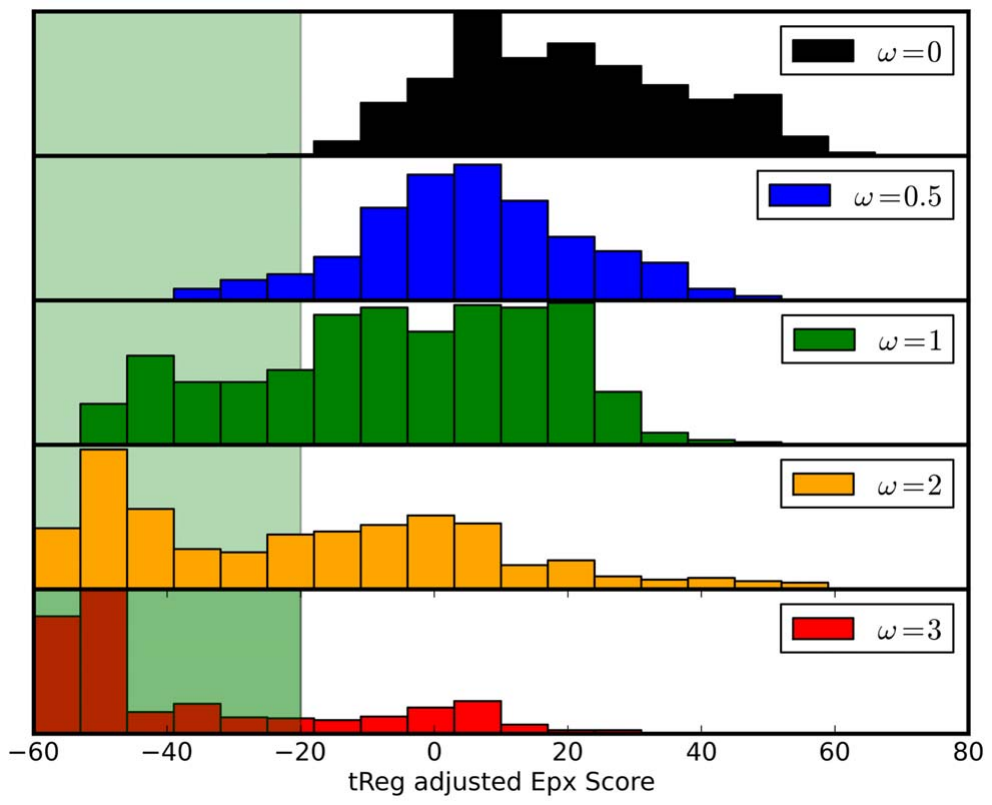
Influence of couplings on generated sequences. The histograms show the distributions of Epivax scores for ensembles of sequences generated with different influence of positional couplings (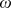). The green shaded area marks the desired region for non-immunogenic sequences.

As it has been outlined before positional couplings in the sequences of antibodies can be regarded as a species-specific fingerprint. If couplings are not considered it consequently should not be possible to sample *human-like* sequences when starting from a mouse sequence. With the introduced humanization protocol this experiment can be done by switching off the coupling terms in the objective function used in the Monte Carlo sampling (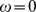 in equation 4). The result of this experiment is shown in figure 6 where the sequence trajectory starting from the Rituximab heavy chains is plotted against the Epivax scores. Although the sequence identities to the parental chain are comparable to those of the sequences generated with the full scoring function, the simulation protocol fails to generate sequences with low predicted immunogenicty. In order to elucidate the useful range of values for omega in a stochastic humanization approach, trajectories from MC samplings (starting from Rituximab-VH) using different values for 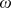 were evaluated with respect to their potential to create low-immunogenic sequences. The histograms in figure 7 show the distributions of Epivax scores for the generated ensembles. With 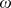-values of 0 and 0.5 sequences in the low immunogenic regions are hardly sampled, whereas increasing 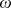 to 1 or 2 yields larger fractions of the desired sequences. Increasing 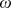 further to 3 constricts the sampled sequence space to a very narrow region around the closest human germline, which consequently is not immunogenic. However, for efficient sampling of a resonable sequence space, 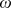-values between 1 and 2 seem to be a good choice.

The consideration of positional couplings for antibody sequence assessment and optimization thus is of utmost importance as they intrinsically reflect the constraints of in-vivo antibody development in a particular species. This is particularly attractive as such constraints are beyond the scope of structure-based optimization methods. If structural information is available, either from modelling or from experimental data, protein design algorithms can be used to probe the effect of mutations on thermodynamic stability or binding affinity [38]–[40]. As the total sequence space which is theoretically available for a variable domain of an antibody is virtually infinite, the Monte-Carlo sequence ensembles can be used to dramatically reduce the degrees of freedom for a structure-based optimization and thereby ensure that only relevant, *native-like* sequences are considered.

Antibody engineering is still mostly an empirically driven discipline as it is notoriously difficult to relate complex processes like expression, shelf-life and PK properties to distinct sequence patterns or structural features. Heuristic scoring functions derived from the sequences of known antibodies offer a smart way to deal with such cases as they do not require a detailed understanding of the underlying physical and biological principles but rather provide an empirical description of what was the result of the complex processes when carried out many times. By analyzing antibody sequence data that have been assembled over decades it is implicitely assumed that sequence patterns which massively compromise important molecule properties occur comparably rare. The scoring functions introduced here were derived from such datasets and are suitable to detect sequence patterns that occur only infrequently in the phenotypic antibody pool of a particular species and which might give rise to manufacturability problems or immunogenicity. Using the score as an objetive function in a Monte Carlo sampling algorithm allows for a stochastic humanization protocol which optimizes humaness and sequence identity to the parental sequence simultaneously, thereby sampling sequences with low predicted immunogenicity.

## Materials and Methods

### Sequence Preparation and Alignment

**Figure 8:**
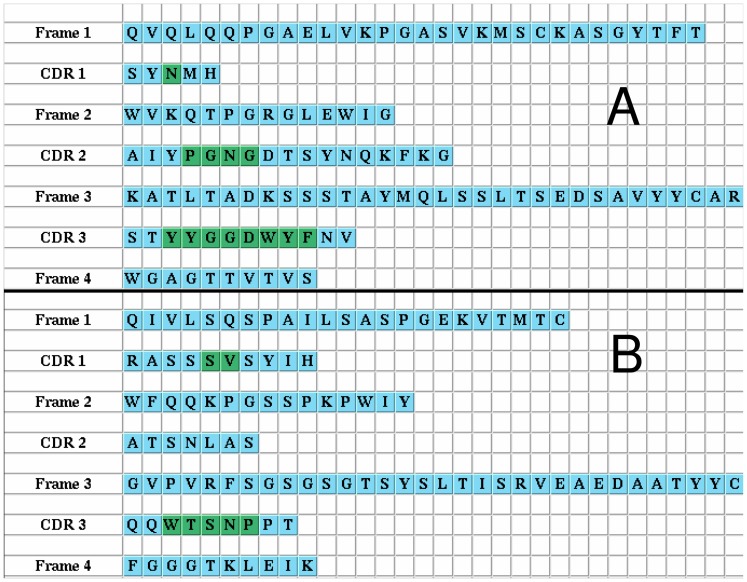
Sequence preparation. Sequence processing is shown for heavy (A) and light chain (B) of Rituximab as an example. CDR residues marked in green belong to structurally varying parts and are not considered in the analysis.

Sequences of heavy and light chains from human and murine antibodies were downloaded from the Abysis database (http://www.bioinf.org.uk/abysis/). For the heavy chain dataset only sequences were selected that are complete from H1 to H112 according to Kabat notation. For light chains residues L1 to L107 were considered. Since the sequence analyses in this paper focus on framework variations, only those parts of the CDRs were taken into account that are structurally conserved which means the begin and the end or, in case of CDR2, also the part which forms defined secondary structure. The residue selection is demonstrated for the heavy and light chains of Rituximab as shown in figure 8. Amino acids shown in green are not considered in the analysis. Processing of the Abysis database and filtering for redundancy (some sequences appear more than once) yielded 5663 unique and complete human heavy chain sequences, 1456 human 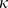-type light chains and 1273 human 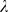-type light chains. For murine sequences 1726 heavy chains, 1636 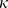-type light chains and 95 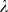-type light chains were obtained. Due to the small number of murine 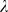-chains no scoring function was derived for this chain type. Multiple sequence aligments (MSA) consisting of the framework residues and the truncated CDRs as described above were generated for each chain type and can be found in (File S1). Since the lengths of the frameworks in antibodies are consereved within the same chain type sequence constructing sequence alignments is trivial. Consequently the alignments do not contain gaps which makes calculation of the frequencies straightforward.

### Computational Tools

Programs for handling of antibody sequences, calculations of scoring functions and Monte Carlo sequence sampling were written in C++. Figures were prepared with the matplotlib library which is part of an inhouse developed antibody analysis software written in C++/Python [41].

## Supporting Information

File S1 **Contains the processed antibody sequences that were used to derive the scoring functions.**
(ZIP)
